# A Real-World Study on the Effectiveness and Safety of Elacestrant in Patients with *ESR1*-Mutated Metastatic Breast Cancer Progressing After CDK4/6 Inhibitors and Endocrine Therapy

**DOI:** 10.3390/cancers18132042

**Published:** 2026-06-24

**Authors:** Martina Greco, Vittorio Gebbia, Rossana Berardi, Antonella Usset, Giuseppina Ricciardi, Nicla La Verde, Maria Vita Sanò, Federica Martorana, Nicoletta Staropoli, Gianfranco Pernice, Gabriella Bini, Angela Prestifilippo, Francesco Giotta, Domenico Bilancia, Calogero Cipolla, Martina De Luca, Maria Rosaria Valerio

**Affiliations:** 1 Medical Oncology Unit and Breast Unit, Policlinico P. Giaccone, University of Palermo, 90100 Palermo, Italy; doc.martinagreco@gmail.com (M.G.); martinamariadeluca@gmail.com (M.D.L.); mariarosaria.valerio@unipa.it (M.R.V.); 2 Medical Oncology, Department of Medicine and Surgery, Kore University of Enna, 94100 Enna, Italy; 3 Medical Oncology Unit, CDC Torina, 90145 Palermo, Italy; 4 Medical Oncology, Università Politecnica delle Marche, AOU delle Marche, 60126 Ancona, Italy; r.berardi@univpm.it; 5 Medical Oncology, Ospedale Civico ARNAS, 90127 Palermo, Italy; antonella.usset@arnascivico.it; 6 Medical Oncology, Ospedale Papardo, 98121 Messina, Italy; giusyricciardi81@hotmail.it; 7 Medical Oncology Unit, Ospedale Sacco, 20157 Milano, Italy; nicla.laverde@asst-fbf-sacco.it; 8 Medical Oncology, Humanitas Istituto Clinico Catanese, 95045 Catania, Italy; maria_vita.sano@humanitascatania.it (M.V.S.);; 9 Department of Clinical and Experimental Medicine, University of Catania, 95123 Catania, Italy; 10 Medical Oncology, Magna Graecia University of Catanzaro, 88100 Catanzaro, Italy; nicolettastaropoli@gmail.com; 11 Medical Oncology Unit, Ospedale Giglio, 90015 Cefalù, Italy; pernicegianfranco@gmail.com; 12 Medical Oncology Unit, Ospedale Villa Sofia-Cervello, 90146 Palermo, Italy; gabriellabini@libero.it; 13 Medical Oncology Unit, Istituto Mediterraneo di Oncologia, 95029 Viagrande, Italy; angela.prestifilippo@gmail.com; 14 Medical Oncology Unit, Istituto Tumori Giovanni Paolo II, 70124 Bari, Italy; f.giotta@oncologico.bari.it; 15 Medical Oncology Unit, Ospedale San Carlo, 85100 Potenza, Italy; domenico.bilancia@ospedalesancarlo.it; 16 Breast Unit, Policlinico, Department of Biomedicine, Neuroscience and Advanced Diagnostics, University of Palermo, 90100 Palermo, Italy; calogero.cipolla@unipa.it

**Keywords:** metastatic breast cancer, ESR-1 mutations, elacestrant, side effects, real world

## Abstract

**Background/Objectives**: Advanced hormone receptor-positive (HR+), epidermal growth factor 2-negative (HER2−) breast carcinoma (BC) patients receive frontline therapy with cyclin-dependent tyrosine kinase 4/6 inhibitors + endocrine therapy (ET). At progression, the best management includes mutational analysis for ESR-1, allowing second-line therapy with elacestrant. The aim of this study was to evaluate the efficacy and safety of elacestrant in an Italian real-world setting. **Methods**: A multicenter, observational study with a mixed retrospective and prospective design was conducted in 13 medical oncology units across Italy. The study population included adult patients with HR+/HER2− locally advanced or metastatic breast cancer with an activating ESR1 mutation documented by liquid biopsy and progressing after at least one line of endocrine therapy containing a CDK4/6 inhibitor. Mutational analysis of plasma was performed using next-generation sequencing with a multigene panel that included ESR1, PIK3CA, AKT, and PTEN. The sample size was calculated according to the two-stage Simon design. Toxicity was classified according to CTCAE version 5.0 criteria. Survival analyses were conducted using the Kaplan–Meier method. **Results**: At the time of analysis, 39 evaluable patients were enrolled, all female and Caucasian, with a median age of 67 years (range 41–89). The efficacy analysis documented an overall ORR of 28% and a disease control rate of 56%. The median duration of response was 6+ months (95% CL: 3.5–10.6 m). Median overall survival was not reached with a median follow-up of 10 months. The toxicity profile was overall favorable: grade ≥2 asthenia was the most frequent adverse event (23%), followed by gastrointestinal toxicity, which was generally mild. No treatment-related toxicity was reported in 64% of patients. Dose reductions were necessary in 15% of cases, while permanent treatment discontinuation due to toxicity occurred in only 4%. **Conclusions**: The results of this Italian multicenter observational study confirm the efficacy and tolerability of elacestrant in HR+/HER2− metastatic breast cancer with *ESR1* mutation, in a real-world context consistent with the data from the pivotal EMERALD study and with real-world data present in the literature.

## Simple Summary

Advanced hormone receptor-positive (HR+), epidermal growth factor 2-negative (HER2) breast carcinoma (BC) patients receive frontline therapy with cyclin-dependent tyrosine kinase 4/6 inhibitors + endocrine therapy. At progression, the best management includes mutational analysis for ESR-1, allowing second-line therapy with elacestrant. This paper reports results of an observational study of elacestrant efficacy and safety in a real-world setting. The results for objective response rate, progression-free survival, and overall survival were within the ranges reported in the registrational studies. The type and severity of adverse events associated with elacestrant were also within the range reported by other authors. Further studies are needed to understand primary resistance to elacestrant.

## 1. Introduction

To date, the first-line treatment for advanced/metastatic hormone receptor-positive (HR+), epidermal growth factor 2-negative (HER2−) breast carcinoma (BC) is represented by cyclin-dependent kinase 4/6 inhibitor (CDK4/6i) plus endocrine therapy (ET), either an aromatase inhibitor (AI) or fulvestrant (FUL) [1,2]. Despite a clear advantage in survival outcomes, HR+/HER2−/BC patients experience disease progression due to the development of resistance to CDK4/6i and standard ET, with a negative effect due to reduced efficacy of subsequent lines of therapy and increased toxicity [3,4]. There are several molecular mechanisms causing primary and secondary endocrine resistance [4,5]. Resistance can develop due to either sub-clonal alterations of the estrogen receptor (ER) pathway, such as the activation of upstream growth factor signaling pathways like the phosphatidylinositol 3-kinase PI3K/AKT/mTOR pathway, or mutations in estrogen receptor 1 (*ESR1*), the latter detected in 40% to 50% of BC patients with progressive disease [6,7,8,9]. These mutations may be effectively targeted with new agents; therefore, major scientific societies recommend testing progressive cases using liquid biopsy to detect ESR1 mutations [1,2].

Elacestrant (ELA) is a recently approved oral selective ER degrader (SERD) that is very active in pretreated HR+/HER2−/BC patients harboring *ESR1* mutations [10,11,12]. The SERD-induced degradation reduces the number of functional ERs in a dose-dependent manner, thereby preventing the transcription of ER-regulated genes that drive cancer growth [13,14]. In cell lines, ELA inhibits estradiol-related functions, including transcription induction of ER genes and cell proliferation, and is also active in preclinical CDK4/6i-resistant xenograft models and those harboring ESR1 mutations [15,16,17,18,19].

Dose-assessment trials employing a single oral dose/day of ELA showed that doses up to 1000 mg/day were safe and well tolerated, with a mean half-life ranging from 27 to 47 h. In murine models, ELA showed a moderate ability to cross the blood–brain barrier, but in humans, central nervous system levels were very low, even though they may be higher in individuals with brain metastases and alterations in the blood–brain barrier [17,20]. The maximum tolerated dose was not reached, but ER occupancy in uterine tissue reached 90% at 500 mg/day. ELA reached steady state after 5–6 days, supporting the use of a single daily dose of 400 mg, as it was well tolerated by most patients [20]. As with all antineoplastic agents, ELA also has some side effects, mainly gastrointestinal, such as nausea, dyspepsia, esophageal spasms and pain, vomiting, and headache. Recent data from a large phase III randomized trial (EMERALD) demonstrated the efficacy of ELA in 477 patients with advanced or metastatic HR+/HER2/BC pretreated with one to two lines of ET, a CDK4/6i, and ≤1 line of chemotherapy [21]. The series included 47.8% of patients with the ESR-1 mutation. PFS was prolonged in all patients (hazard ratio—HR—0.70; 95%CI 0.55–0.88; *p* = 0.002) and patients with *ESR1* mutation (HR 0.55; 95%CI 0.39–0.77; *p* = 0.0005).

In this paper, the authors report real-world data on the efficacy and safety of ELA in HR+/HER2−/BC patients progressing after one or more endocrine lines of treatment, including CDK4/6i.

## 2. Materials and Methods

**Figure 1:**
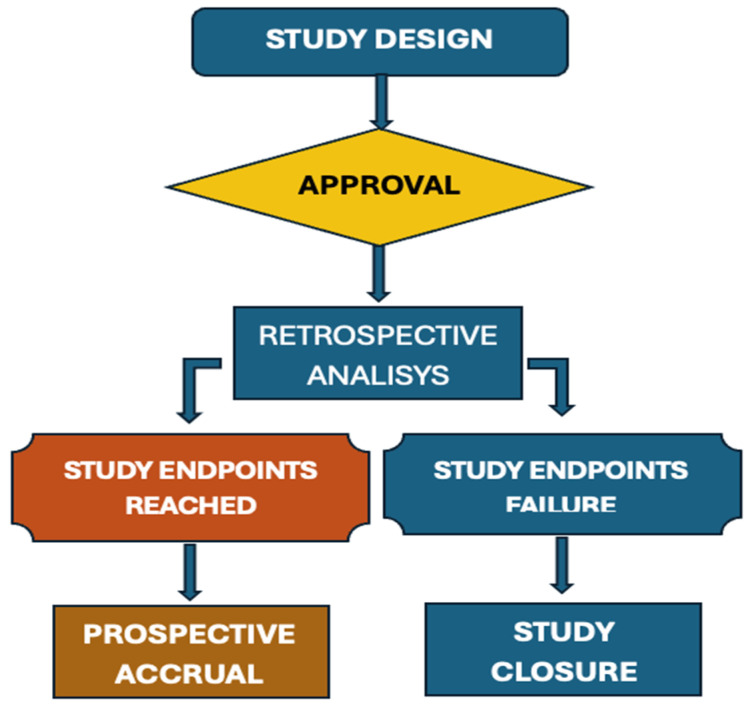
Study outline.

Study Design. This study is a multicentric mixed retrospective and prospective analysis of real-life patients with hormone-sensitive, HER2-negative metastatic BC who experienced disease progression after CDK4/6i-based therapy. All patients were screened for ESR1 and PIK3CA/AKT/PTEN mutations. Mutated patients treated with ELA were included in the final analysis. The study protocol was approved by the Ethics Committee Palermo-1, Policlinico, University of Palermo. Figure 1 shows the outline of the study.

Treatment. ELA monotherapy was prescribed according to the guidelines of the Italian Agency for Drugs (AIFA) and the European Medicines Agency (EMA) for patients with ER-positive, HER2-negative, locally advanced or metastatic breast cancer with an activating ESR1 mutation employing a validated test who showed disease progression after at least one line of ET, including a CDK4/6i [22]. ELA was administered at the recommended dose of 345 mg daily until clinical benefit was observed or unacceptable toxicity occurred. If a dose is missed, it can be taken immediately as long as no more than 6 h have passed since the scheduled time. If more than 6 h have passed, the dose should be skipped for that day. The next day, ELA should be taken at the regular time. For all drugs, dose modifications were made by treating oncologists as needed according to the published guidelines [22]. Treatment schedule included ELA 345 mg/day until progression, severe toxicity, or refusal to withdraw from the study. In case of a missed dose, ELA may be taken within 6 h. according to the drug’s pharmacokinetics.

Accrual criteria. Entry criteria included age >18 years, performance status of 0–2 according to the ECOG scale, locally advanced and/or metastatic BC, measurable disease according to the RECIST criteria with at least one bone lytic lesion, adequate bone marrow function, and no severe or uncontrolled diseases other than cancer. Exclusion criteria were a known allergy to the same class of agents, visceral crisis, previous radiotherapy completed less than 4 weeks before starting ELA (except for short-term analgesic treatment for bone pain), severe cardiovascular disease, and gastrointestinal diseases. All patients had to have previously received ≥12 months of CDK4/6i and had clinically or radiologically documented disease progression. Patients were also required to provide informed consent to start therapy and for data extraction. In the event of clinically meaningful toxicity, the ELA dose was adjusted according to the published recommendations [22]. Drug–drug interaction checking was performed to assess the need to avoid concomitant use of strong or moderate CYP3A4 inhibitors and to identify an alternative concomitant medication with no or minimal CYP3A4 inhibitory potential. If a potent CYP3A4 inhibitor is necessary, the ELA dose should be reduced to 86 mg once daily, with close monitoring of tolerability. If a mild CYP3A4 inhibitor is necessary, the ELA dose should be reduced to 172 mg once daily, with close monitoring of tolerability. A subsequent decrease to 86 mg daily may be contemplated with mild CYP3A4 inhibitors, contingent upon tolerability.

NGS analysis. The search for ESR-1 and PI3KCA/AKT/PTEN mutations was performed using a liquid biopsy. Briefly, Plasma samples from patients with metastatic BC were obtained by drawing approximately 15 mL of whole blood into EDTA-containing Vacutainer tubes. Within one hour of collection, the samples were centrifuged to separate the plasma component, which was then aliquoted and stored at −80 °C until analysis. Circulating cell-free DNA (cfDNA) was extracted from the plasma using an automated magnetic bead-based method and quantified by fluorimetry. NGS analysis was performed using a targeted (amplicon-based) multigene panel that included key predictive genes for therapeutic response, such as ESR1, PIK3CA, AKT, and PTEN. Data processing was performed using the manufacturer’s software, followed by quality verification via visual inspection of the raw file on dedicated bioinformatics platforms.

Sample size. The sample size was determined using an optimal two-stage Simon’s design. The median objective response rate (13%) reported in the medical literature was used as the cutoff endpoint, and the design was configured with a type I error rate of 0.05 and 80% power. The null hypothesis was that the true response rate was 0.01, while the alternative hypothesis was that it was 0.15. In stage I, 11 patients needed to be enrolled. If no responses are observed among these 11 patients, the study will be stopped early. Otherwise, an additional 15 patients will be enrolled in stage II, bringing the total to 26. If two or more responses are observed among these 26 patients, the null hypothesis will be rejected, and the treatment will be considered promising and compared to results published in the medical literature at the time of the analysis.

Outcome evaluation. Staging of disease was based on physical examination, CT or PET scan, sonograms, or NMR as needed. Objective response rate (ORR) and progression-free survival (PFS) were the principal study endpoints; secondary endpoints included safety, duration of response, and overall survival (OS). Objective responses were reported according to the RECIST criteria [23]. Briefly, CR was defined as “the disappearance of all target lesions and the reduction of any pathological lymph nodes in the short axis to <10 mm,” and PR as a >30% decrease in the sum of diameters of target lesions. Stable disease (SD) was defined as neither sufficient shrinkage to qualify for PR nor sufficient increase to qualify for PD, with the smallest sum of diameters in the study as the reference, and progressive disease (PD) as at least a 20% increase in the sum of diameters of target lesions. In addition to the relative increase of 20% without the appearance of one or more new lesions.” The median duration of objective response was calculated from the date of first administration of ELA until the date of disease restaging showing progression or the last known follow-up visit. PFS was calculated from the start of ELA administration to the onset of clinical or radiological disease progression.

OS was calculated from the beginning of ELA until death from any cause or the last follow-up. Evaluation of the response was performed every 3 months or as clinically needed. PFS and OS were calculated from the date of first ELA treatment to the date of clinical or radiographic progression, death, or last known follow-up. The Common Terminology Criteria for Adverse Events (CTCAE) version 5 was used to grade and report side effects [24].

Statistics. Quantitative data on patients’ demographic and clinical characteristics were reported as absolute numbers, with percentages rounded to the nearest whole number, and 95% confidence limits (95% CL). Percentages were rounded to the nearest unit. A chi-square test was applied to a contingency table to compare quantitative data. Time-to-progression and overall survival were computed using the Kaplan–Meier method in GraphPad Prism 11 (GraphPad Software, 225 Franklin Street. Fl. 26, Boston, MA, USA). A meta-analysis was performed using a classical fixed-effects model. Investigators employed Cohen’s kappa coefficient to measure the inter-rater degree of agreement on selected papers [25].

## 3. Results

### 3.1. Patients’ Population

The study accrued 39 patients screened for mutational status enrolled at 13 medical oncology units in Southern Italy. The latter included 4 academic hospitals, 12 cancer centers, and 8 general or community hospitals. Table 1 depicts the main clinical and demographic characteristics of enrolled patients. All patients were Caucasian females. Briefly, patients had a median age of 67 (range, 41–89) and a median ECOG PS of 1 (range, 0–2). Patients had a luminal A or B carcinoma in 71% and 29% of cases, respectively. The main sites of disease were the liver, lungs, nodes, and bones. All patients treated with ELA had previously received therapy with CDKI4/6i with AI or FUL. All patients showed progressive disease when ELA was prescribed. Genomic alterations were *ESR1* alone or PIK3CA + ESR1 in 33 (84%) and 6 (16%) cases, respectively.

### 3.2. Clinical Outcomes

**Figure 2:**
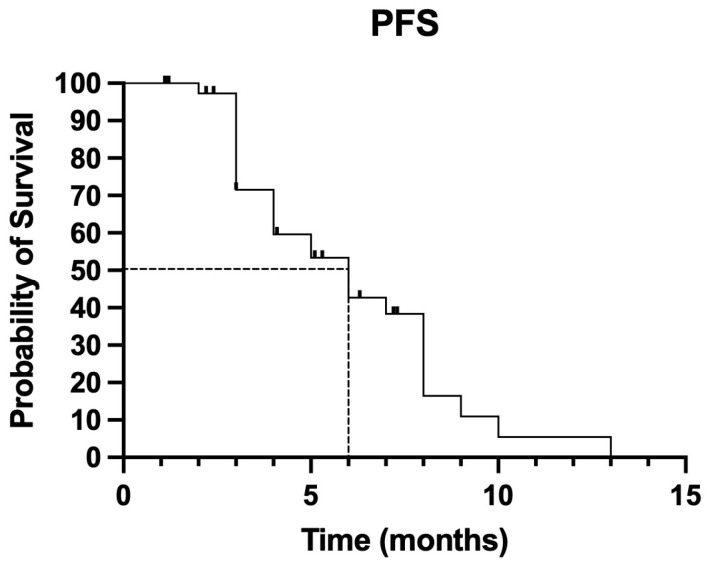
Progression-free survival of the whole series of patients (black squares indicate censored patients).

**Figure 3:**
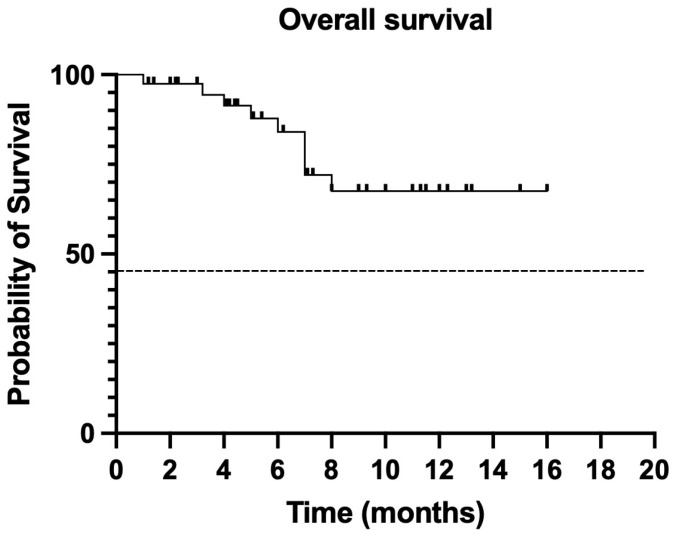
Overall survival of the whole series of patients (black squares indicate censored patients).

Table 2 shows the response rates achieved in the present study and those reported in the medical literature for the same clinical setting. In our study, 1 patient (2%) experienced a CR, 10 patients (26%) had a PR with a median duration of 6.5 months, 11 patients (28%) had disease stabilization, and 12 patients (31%) showed progressive disease. As shown in Table 2 and Figure 2, median PFS was 6.0+ months (95% CL 4.0–8.0) and median OS was not reached after a median follow-up of 8.5 months. Figure 3 shows a meta-analysis of objective response rates of the main published studies. The analysis showed ORR data slightly better but compatible to other scientific reports. PFS mature data are awaited to perform metanalysis.

### 3.3. Safety

Table 3 shows the main side effects according to NCCN CTC version 4.1. Grade 3 fatigue, cutaneous, and gastrointestinal AEs were observed in 2% of cases. Any grade fatigue was reported in 35% of patients. In two patients, fatigue and skin toxicity caused treatment withdrawal. No grade 3 hematological toxicity was observed, and anemia was the most frequent blood count AE. Overall, 6 patients (15%) required dose reduction.

## 4. Discussion

Despite the undeniable progress achieved in the last decade, the clinical outcomes of patients with advanced or metastatic HR+/HER2−/BC progressing after previous lines of therapy, including a CDK4/6i, remain far from satisfactory [21,26]. Today, these progressing patients are best managed with new oral selective estrogen receptor degraders (SERDs) based on mutation analysis [27,28]. A pooled analysis of the four randomized clinical trials ACELERA, AMEERA-3, EMERALD, and SERENA-2, including 1290 patients on oral SERDs, showed a PFS advantage when compared with treatment of physician’s choice (HR 0.783, 95%CI 0.681–0.900, *p* < 0.001), which was higher in the *ESR1* mutated subgroup (HR 0.557, CI 0.440–0.705, *p* < 0.001) [29]. On the other hand, oral SERDs showed no PFS benefit in ESR1 wild-type patients (HR 0.944, 95% CI 0.783–1.138, *p* = 0.543) compared with treatment of the physician’s choice.

Among SERMs, ELA has been the most investigated and is available for prescription in clinical practice, approved based on phase I and randomized trials for the treatment of progressing HR+/HER2−/BC harboring the *ESR1* mutation. An international phase Ib trial on 16 postmenopausal women with HR+/HER2−/BC progressing after 1–3 lines of endocrine treatment who received oral daily ELA reported an 89.1% median reduction of 16α-18F-fluoro-17β-estradiol positron emission tomography with low-dose computed tomography showing a greatly reduced ER availability, which, however, did not correlate with the 11% ORR and 30.8% clinical benefit rate (CBR) [30]. In 2021, Bardia et al. reported results of a phase I study of ELA in a series of 63 postmenopausal women with heavily metastatic HR+/HER2−/BC pretreated with a median of three lines of therapy [31]. Half of the patients harbored the ESR-1 mutation and had previously received CDK4/6i and FUL. The most frequent toxic effects included nausea, dyslipidemia, and hypophosphatemia, leading to a recommended phase II dose of 400 mg once daily, even if a maximum tolerated dose was not reached. The ORR was 19.4% across the entire series, 33% in ESR1-Mutant patients, 15.0% in patients with prior SERD, and 16.7% in patients pretreated with CDK4/6i. Among the 47 patients treated with the recommended dose, the clinical benefit rate was 42.6%, ranging from 56.5% in the ESR-1-Mutant cohort to 30.4% in those with prior CDK4/6i therapy. Interestingly, a decrease in ESR-1-mutant allele frequency was associated with CBR.

ELA efficacy has been evaluated in the randomized phase III EMERALD clinical trial (NCT03778931), where it was compared with the standard of care in nearly 500 patients with advanced HR+/HER2−/BC. Even though median PFS was 2.8 and 1.9 months in the ELA and standard-of-care arms, respectively, ELA reduced the risk of PFS by 30%. In the cohorts with ESR1 mutations, PFS was 3.8 and 1.9 months in the ELA and standard-of-care arms, respectively, with a 45% risk reduction [21]. Overall, these results are statistically significant, but the overall gain was small, highlighting the limits of mono ET in patients with BC progressing after ET and CDK4/6i. As reported by Shah et al. in 2023, the EMERALD trial showed a statistically significant improvement in PFS (HR 0.55; 95% CI 0.39–0.77; *p* = 0.0005) among ESR1-mutant patients [27]. Although the OS endpoint was not reached, there was an acceptable safety profile without a trend toward a potential OS detrimental effect (HR 0.90; 95%CI, [0.63–1.30]) in the ESR1-mutant patients. PFS also reached statistical significance in the intention-to-treat (ITT) population (HR 0.70; 95% CI 0.55–0.88; *p* = 0.0018). The authors felt that the improvement in PFS in the ITT population was primarily attributable to the results observed in ESR1-mutant patients. Subsequently, Bardia et al. reported a subgroup analysis of ELA activity in a cohort of 159 patients with an ESR-1 mutation who were pretreated with ET plus CDKi4/6 for at least 12 months [32]. In this favorable population, the median PFS was 8.61 (range 4.14–10.84) versus 1.91 (range 1.87–3.68) months for ET plus ELA versus FUL (HR 0.41; 95%CI 0.26–0.63). The median PFS for ELA versus the standard of care was 9.1 months versus 1.9 months in patients with bone metastases, 7.3 versus 1.9 months in patients with liver and/or lung metastases, 9.0 versus 1.9 months in those with <3 metastatic sites, and 10.8 versus 1.8 months in cases with ≥3 metastatic sites. Moreover, median PFS was 5.5 versus 1.9 months in patients with PIK3 catalytic subunit α mutation, 8.6 versus 1.9 months in patients with tumor protein p53 gene mutation, 9.0 versus 1.9 months in the HER2-low group, 9.0 versus 1.9 months in patients with ESR1D538G-mutated tumors, and 9.0 versus 1.9 months in those harboring the ESR1Y537S/N-mutation. Subgroup safety was consistent with the overall population [32]. A cost-effectiveness analysis of pretreated advanced or metastatic ER+/HER2− BC treated in the EMERALD trial in the USA showed that second- and third-line ELA was not cost-effective when compared to the standard of care [33].

**Figure 4:**
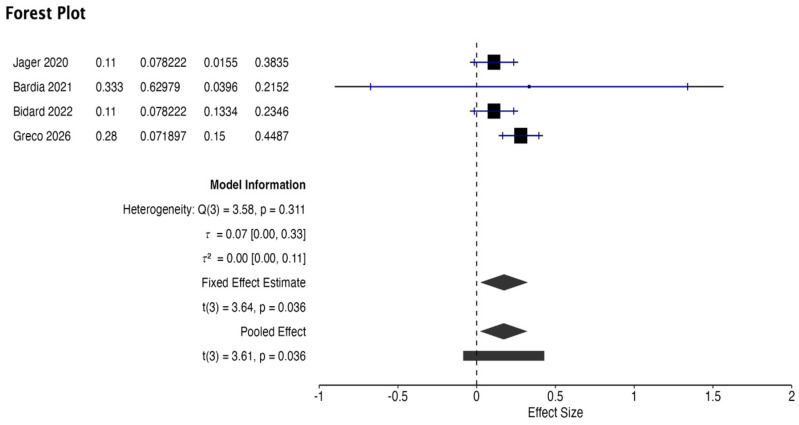
Objective response meta-analysis of currently published studies [21,30,31].

Safraz et al. conducted a pooled study of PFS with a random-effects model, yielding an estimated value of 4.38 (95% CI, 7.58–16.35) [34]. The heterogeneity analysis indicated τ2 = 0, and the Q-value from the heterogeneity test was 0.11 with 2 degrees of freedom (*p* = 0.94), implying an absence of heterogeneity among the studies for PFS (Figure 4). The results were not statistically significant (z = 0; *p* = 0.47), suggesting that the intervention did not meaningfully affect PFS. The pooled analysis ORR was 7% (95% CI 2–18%) according to the random-effects model. The heterogeneity analysis indicated significant variability among the trials, with a τ2 of 0.5749, an H of 1.78 (95% CI 1.00–3.30), and an I2 of 68% (95% CI 0.0–90.8%). The Rb value was 63.6% (95% CI 4.7–100.0%). The heterogeneity test produced a Q-value of 6.33 with 2 degrees of freedom (*p* = 0.04), indicating considerable variability for ORR across the included studies.

ELA has also been tested in 22 patients in a preoperative setting [35]. This agent induced a complete cell cycle arrest in 27% of cases and a reduction in Ki-67 of 52.9%. Tumor phenotyping by the PAM50 signature showed increased activation of immune-response genes and suppression of proliferation and estrogen-related genes, including ESR-1, indicating a shift toward a more endocrine-sensitive biological profile.

Lloyd et al. conducted a real-world study of ELA and clinical–genomic factors associated with clinical outcomes in 756 patients (76% pretreated with CDK4/6i and 38% with chemotherapy) who had activating ESR1 mutations detected <6 months before ELA initiation [36]. The median time-to-treatment-discontinuation and median time-to-next-treatment (TTNT) were 4.6 and 6.4 months, respectively; the latter was longer in patients treated with fewer than 1 line of therapy for advanced disease than in those receiving third-line therapy (8.8 versus 6.0 months). Patients pretreated with FUL and higher ESR-1 polyclonally had a trend toward shorter treatment duration, but efficacy was consistent across ESR1 alleles (e.g., Y537S and D538G). In diseases with dual mutations in the ESR-1 and PI3K pathways, ELA showed activity comparable to that observed in phase III studies.

In a very recent real-world analysis of 306 patients with advanced HR+/HER2−/BC and ESR1 mutations treated with ELA, 93.8% had received prior ET ± CDK4/6i for ≥12 months, 50.0% had received chemotherapy, and 72.2% had received FUL [37]. The median time to next treatment (mTTNT) was 7.9 months in the overall population, with values of 8.2 months in patients with 1–2 prior lines and 7.5 months in those with ≥3 prior lines. Even in tumors with co-mutations in ESR1 and the PI3K pathway, mTTNT remained clinically meaningful (6.3 months). The benefit was maintained in clinically relevant subgroups, including patients without prior FUL (12.9 months), without prior chemotherapy (8.4 months), with visceral metastases (7.9 months), and with liver metastases (7.2 months). Overall, ELA demonstrated sustained benefit in real-world clinical practice, supporting its use in the personalized sequencing of endocrine therapy prior to targeted chemotherapy or combination therapy.

The safety profile of ELA has been considered acceptable by most agencies. In the EMERALD trial, patients receiving ELA infrequently experienced adverse events (AEs) that led to treatment discontinuations (6.3%), comparable to the standard-of-care groups (4.4%) [21,27,32]. Although ELA generally has a favorable safety profile, it may cause AEs such as nausea, vomiting, disease progression, elevated liver enzymes, hot flashes, and, most importantly, dehydration, abnormal tumor markers, and esophageal-related reactions [21,27,38,39,40]. The latter have been hypothesized to be linked to its metabolites, the rapid drug release from pills, the number of pills, and their formulation as tablets, which was more tolerated than capsules [15,20,38,39,40,41]. The monoclonal antibody cancer antigen 27.29 targets the glycoprotein MUC1 expressed on the apical surface of normal epithelial cells. Approximately two-thirds of individuals with advanced illness exhibit this antigen [42]. The mechanism responsible for the ELA-induced increase in tumor markers may be attributed to its rapid promotion of tumor cell death, leading to the release of specific markers from dying tumor cells into the bloodstream, causing a temporary rise in serum biomarker levels. A pharmacovigilance study employing the Food and Drug Administration Adverse Event Reporting System (FAERS) confirmed that nausea, vomiting, fatigue, dyslipidemia, and musculoskeletal pain were the more common, well-known side effects [40]. Moreover, this study reported some potentially new AEs, including lymphedema, bone-related events, reflux esophagitis, hypokalemia, and dehydration [40]. The median time to toxicity was 44 days from the beginning of treatment. Post-marketing analysis of AEs reported progressive tumors, drug ineffectiveness, dose omissions, arthralgia, asthenia, and bone pain as the most frequent AEs not reported in the drug label [39]. Safety analysis reported gastrointestinal disorders and musculoskeletal and connective tissue disorders as the most frequent complaints.

Figure 4 depicts objective response meta-analysis of currently published studies. Data reported in the present study confirm the results of the registrational trial and other published series of patients in a real-life, multicenter setting. The safety profile of ELA was acceptable, with no treatment withdrawal due to patients’ refusal to severe toxicity. No severe hypoglycemia was seen. Table 4 shows the main studies published in medical literature. In our study, the objective response rate of 28.1% (95% CI 0.15–0.4487) was superior to those reported by other authors, with 95% CI values within the range reported in the medical literature. The ORR data could be explained by favorable patient entry criteria, such as the duration of prior CDK4/6i treatment and disease burden.

Correlation with clinical data, such as previous response to CDK4/6i, median PFS from previous therapy, disease sites, or other factors, is pending until PFS results are more mature. Median PFS of 6.0+ months (95% CI, 4.0–8.8) is lower than that reported in patients with >12 months of prior CDK4/6i treatment (8.61 months; 95% CI, 4.1–10.84). The presence of many patients with censored PFS below the median may explain this difference. Our study, however, presents several limitations intrinsic to its partially retrospective nature. Among these limitations are the possible underestimation or underreporting of toxicity, which may be due in part to oncologists’ short learning curves. The geographic locations of participant centers may not represent the full national landscape. The patient population is Caucasian, which may limit the generalizability of the findings to a broader global population. Lastly, the heterogeneity in the clinical characteristics of enrolled patients may confound the interpretation of the results. In all reported series, including the present one, the PFS curves show that nearly 35% of patients progress within the first 3 months after the start of ELA, suggesting the coexistence of other, currently not fully elucidated, mechanisms of resistance that reduce sensitivity to ELA. The estrogen receptor exists in two major isoforms, ERα and ERβ, the latter of which has an unclear role in cancer and is encoded by different genes on chromosomes 6 and 14, respectively, which regulate distinct pathways [43,44,45,46]. Therefore, further biomolecular and clinical research in this setting is eagerly needed.

Testing for *ESR1* mutations is pivotal for selecting the best treatment and guiding therapy with novel SERMs, and liquid biopsy is the recommended test for mutation detection, as employed in most studies [47,48]. The emergence of mutations conferring ET resistance during CDK4/6i-based treatment is a dynamic process; therefore, a low-invasive and repeatable test such as liquid biopsy has clear practical advantages, even if the timing has yet to be established [49,50,51]. However, next-generation sequencing (NGS) was performed on formalin-fixed paraffin-embedded (FFPE) tissue biopsy samples from metastatic sites at the time of disease progression after ET and CDK4/6i [52]. ESR1 mutations were detected in 63.2% of 38 patients, with p.D538G and p.Y537S being the most frequent alterations. ESR-1-mutant cases showed a higher incidence of lung metastases than wild-type cases (33.3% versus 7.1%). Interestingly, a dual ESR1 mutation and a recurrent ESR1-CCDC170 gene fusion were identified, along with co-mutations in the PIK3CA pathway (41.6%), suggesting a more complex interplay in the mechanism of resistance and the potential usefulness of combined treatments.

The efficacy of SERMs has also been shown in the adjuvant setting, further supporting this class of drugs. Very recently, a large phase III trial randomized 4170 patients to giredestrant or standard-of-care ET, showing superior invasive disease-free survival with the former, with an HR of 0.70 (95% CI 0.57–0.87; *p* = 0.0014) [53]. In both arms, the most common AEs were arthralgia (48.0% versus 47.1%), hot flush (27.4% versus 28.8%), and headache (15.3% versus 13.2%), while the most common grade 3–4 AEs were hypertension (2.6% versus 2.0%) and arthralgia (1.5% versus 1.8%). The discontinuation rate due to AEs was lower with giredestrant (5.3%) than with standard-of-care ET (8.2%).

## 5. Conclusions

Our real-life study confirms the efficacy and safety of ELA, as reported in the registrational study and other series in the medical literature. Further analysis will be performed as the data mature. Despite the observed efficacy of SERMs in advanced HR+/HER2−/BC, a significant proportion of patients do not respond to this approach, and the median duration of clinical benefit remains suboptimal. Therefore, further studies are needed to clarify the mechanisms underlying resistance to currently available agents.

## Figures and Tables

**Table 1:** Main clinical and demographic characteristics of enrolled patients.

Number of Patients		39 (100%)
**Age (years)**	Median (range)	67 (41–89)
**PS (ECOG scale)**	0	16 (41%)
	1	23 (59%)
**Pathology**	No Special Type	34 (87%)
	Lobular	5 (13%)
	Luminal A	28 (71%)
	Luminal B	11 (29%)
**Disease sites**	Bone	36 (92%)
	Liver	23 (58%)
	Node	22 (56%)
	Brain	1 (3%)
	Other	7 (18%)
	Lung	10 (25%)
Previous therapy *	Palbociclib	19 (49%)
	Ribociclib	15 (38%)
	Abemaciclib	9 (23%)
	AI	27 (69%)
	Fulvestrant	12 (31%)
**Genomic alterations**	*ESR1*	33 (84%)
	*PIK3CA* + *ESR1*	6 (16%)

* Patients may have received more than one prior line.

**Table 2:** Objective response rates and survival outcomes.

**Objective Response**	**N. of Patients (Percent)**
Complete response	1 (2%)
Partial response	10 (26%)
Stable disease	11 (28%)
Overall response	11 (28%)
Progressive disease	12 (31%)
**Survival outcomes**	
Median duration of response (months)	6.5 months (range 3–10)
Median duration stable disease	6.0 months (range 2–9)
Median PFS (months)	6.0+ months (range 3.5–10.6)
Median OS (months)	Not reached after median follow-up of 8.5+ months

**Table 3:** Main toxic effects according to the NCCN-CTC version 5.0 (percentages are rounded to the nearest unit).

Toxicity		Grade 1	Grade 2	Grade 3
Hematological	Anemia	4 (10%)	1 (2%)	--
	Neutropenia	2 (4%)	1 (2%)	--
	Thrombocytopenia	1 (2%)	2 (4%)	--
Gastrointestinal	Nausea	5 (13%)	2 (4%)	1 (2%)
	Vomiting	3 (8%)	3 (8%)	1 (2%)
	Diarrhea	2 (5%)	3 (8%)	1 (2%)
Cutaneous		1 (2%)	--	1 (2%) *
Fatigue		5 (13%)	8 (21%) *	1 (2%) *
Dose reduction	6 (15%)			
Treatment Discontinuation	2 (4%)			
Not reported toxicity	25 (64%)			

* Toxicity causing treatment withdrawal.

**Table 4:** Main trials included in the study.

Reference	Sample Size	Age Median	Study Type	Prior Lines	ORR, DTC	PFS/TTNT Months Median	SAEs Grade 3
Jager et al., 2020 [30]	16	57	Phase 1b	No previous CDKI	11%	5.5	Esophagitis 6.3% Anemia 6.3% Lung embolism 6.3% Collapse 6.3% Cystitis 6.3%
Bardia et al., 2021 [31]	57	63	Phase I	CDKI 52% m-Tor. 28% CT 42%	33.3% Duration 5.8 CRB 52%	4.5 Prior CDKI 3.7 No-prior CDKI 7.4	ALT 4%, AST 8% Hypophosphatemia 6% Hyperglycemia2% Vomiting 4%
Bidard et al., 2022 [21]	239	63	Phase III	CDKI 100% ET 100% CT 23%	11% PR 49.4% SD	3.78 ESR1 mutated 6-mos 40.8%	ALT 2% Nausea 2.5% Vomiting 0.8% Back pain 2%
Bardia et al., 2024 [32]	159	65	Emeral trial subgroup analysis	CDK4/6i ET CT	Not reported	8.6	Nausea 2.5% Vomiting 0.8% Abdominal pain 1% Musculoskeletal pain/% Headache 2%
Lloyd et al., 2026 [36]	418	62	Real-world retrospective	CDKI 73% FUL 63% CT 49%	Not reported	TTD 5.4 TTNT 6.2	Not reported
Rugo et al., 2025 [37]	306	64	Real-world retrospective	CDKI 89% FUL 72.2% CT 50%	Not reported	7.9 TTNT 10.8	Not reported

## Data Availability

The raw data supporting the conclusions of this article will be made available by the authors on request.

## Abbreviations

The following abbreviations are used in this manuscript:

ESR-1	Estrogen receptor-1
ORR	Overall response rate
PFS	Progression-free survival
TTNT	Median time to next treatment
OS	Overall survival
